# The Trends of Complicated Acute Colonic Diverticulitis—A Systematic Review of the National Administrative Databases

**DOI:** 10.3390/medicina55110744

**Published:** 2019-11-16

**Authors:** Roberto Cirocchi, Georgi Popivanov, Alessia Corsi, Antonio Amato, Riccardo Nascimbeni, Rosario Cuomo, Bruno Annibale, Marina Konaktchieva, Gian Andrea Binda

**Affiliations:** 1 Department of General Surgery and Surgical Oncology, Hospital of Terni, University of Perugia, 05100 Terni, Italy; roberto.cirocchi@unipg.it; 2 Department of Surgery, Military Medical Academy, ul. “Sv. Georgi Sofiyski” 3, 1606 Sofia, Bulgaria; 3 Chirurgia Generale, Ospedale della Media Valle del Tevere, via del Buda, 06059 Todi, Italy; alessia.corsi2015@libero.it; 4 Unit of Coloproctology, Department of Surgery, Borea Hospital, 18038 Sanremo, Italy; ab.amato@libero.it; 5 Department of Molecular and Translational Medicine, University of Brescia, 25121 Brescia, Italy; riccardo.nascimbeni@unibs.it; 6 Gastroenterology and Digestive Endoscopy Unit, Sant’Anna e San Sebastiano Hospital, 81100 Caserta, Italy; rcuomo67@gmail.com; 7 Department of Medical Surgery, Sciences and Translational Medicine University Sapienza, 00189 Rome, Italy; bruno.annibale@uniroma1.it; 8 Department of Gastroenterology and Hepatology, Military Medical Academy, ul. “Sv. Georgi Sofiiski“ 3, 1606 Sofia, Bulgaria; marina.konaktchieva@yahoo.com; 9 Colorectal Surgery, BioMedical Institute, 16157 Genova, Italy; gabinda@me.com

**Keywords:** complicated acute colonic diverticulitis, temporal trends, hospitalization, national databases

## Abstract

*Background and Objectives*: The diverticular disease includes a broad spectrum of different “clinical situations” from diverticulosis to acute diverticulitis (AD), with a full spectrum of severity ranging from self-limiting infection to abscess or fistula formation to free perforation. The present work aimed to assess the burden of complicated diverticulitis through a comparative analysis of the hospitalizations based on the national administrative databases. *Materials and Methods*: A review of the international and national administrative databases concerning admissions for complicated AD was performed. *Results*: Ten studies met the inclusion criteria and were included in the analysis. No definition of acute complicated diverticulitis was reported in any study. Complicated AD accounted for approximately 42% and 79% of the hospitalizations. The reported rates of abscess varied between 1% and 10% from all admissions for AD and 5–29% of the cases with complicated AD. An increasing temporal trend was found in one study–from 6% to 10%. The rates of diffuse peritonitis ranged from 1.6% to 10.2% of all hospitalizations and 11% and 47% of the complicated cases and were stable in the time. *Conclusions*: The available data precluded definitive conclusions because of the significant discrepancy between the included studies. The leading cause was the presence of heterogeneity due to coding inaccuracies in all databases, absence of ICD codes to distinguish the different type of complications, and the lack of coding data about some general conditions such as sepsis, shock, malnutrition, steroid therapy, diabetes, pulmonary, and heart failure.

## 1. Introduction

The diverticular disease (DD) includes a broad spectrum of different “clinical situations” from diverticulosis to diverticulitis. Diverticulosis means that diverticula is only present at the colonic level, and many patients with colonic diverticula remain asymptomatic throughout their life. Only 20% of patients develop symptoms and signs of illness [1]. If these pouches become inflamed or infected and cause severe abdominal pain and fever, the disease is referred to as acute diverticulitis (AD). AD represents 4% of abdominal pain in patients evaluated at emergency departments [2]. AD could remain uncomplicated in 75% of patients, while approximately 25% will develop complications with a broad spectrum of severity, ranging from mild self-limiting infection to bleeding, abscess, or fistula formation to free perforation [3]. Consequently, diverticulitis is subdivided into uncomplicated and complicated [1].

Some recent studies analyzed the nationwide trend in hospital admissions for complicated AD and its association with gender, age, and type of treatment, including in-hospital mortality, showing an increased rate of AD [4,5,6]. The present study aimed to ascertain the burden of complicated diverticulitis on the healthcare systems by performing a comparative analysis of the international data of the epidemiology of hospitalizations for complicated AD through a systematic review of the literature. The ascertainment of the real incidence of AD (per 100,000) was beyond our scope.

## 2. Materials and Methods

We performed an analysis of the international and national administrative databases concerning admissions for complicated AD. The criteria of the “Preferred Reporting Items for Systematic Reviews and Meta-analyses (PRISMA) statement” were applied [7].

Inclusion criteria: Non-national or regional databases were excluded. An analysis of the national databases was performed with a focus on the acute complicated diverticulitis. No language restrictions were imposed.

Exclusion criteria: Randomized trials and articles that only treated diverticulosis or uncomplicated diverticulitis alone, or with only patients undergoing emergency/emergent surgery for AD, were excluded.

Sources of information: The following shows the systematic search performed on PubMed for papers published from January 1978 to June 2019: (“surgery”[Subheading] OR “surgery”[All Fields] OR “surgical procedures, operative”[MeSH Terms] OR (“surgical”[All Fields] AND “procedures”[All Fields] AND “operative”[All Fields]) OR “operative surgical procedures”[All Fields] OR “surgery”[All Fields] OR “general surgery”[MeSH Terms] OR (“general”[All Fields] AND “surgery”[All Fields]) OR “general surgery”[All Fields]) AND (“diverticulitis”[MeSH Terms] OR “diverticulitis”[All Fields]) AND (“emergencies”[MeSH Terms] OR “emergencies”[All Fields] OR “emergency”[All Fields]). Two other searches were performed on SCOPUS and WOS. Duplicates were filtered and remaining papers were screened for title and abstract. Randomized trials, articles not reporting on the complicated diverticular disease, and those reporting on only emergency surgery were excluded. Expert opinions, reviews, and case reports were also excluded. No language restrictions were applied.

A full-text analysis of the remaining papers was conducted. Only those focusing on the epidemiology of complicated diverticulitis were included in the present review. A final hand-search of references of all included articles was carried out for further relevant studies.

## 3. Results

**Figure 1:**
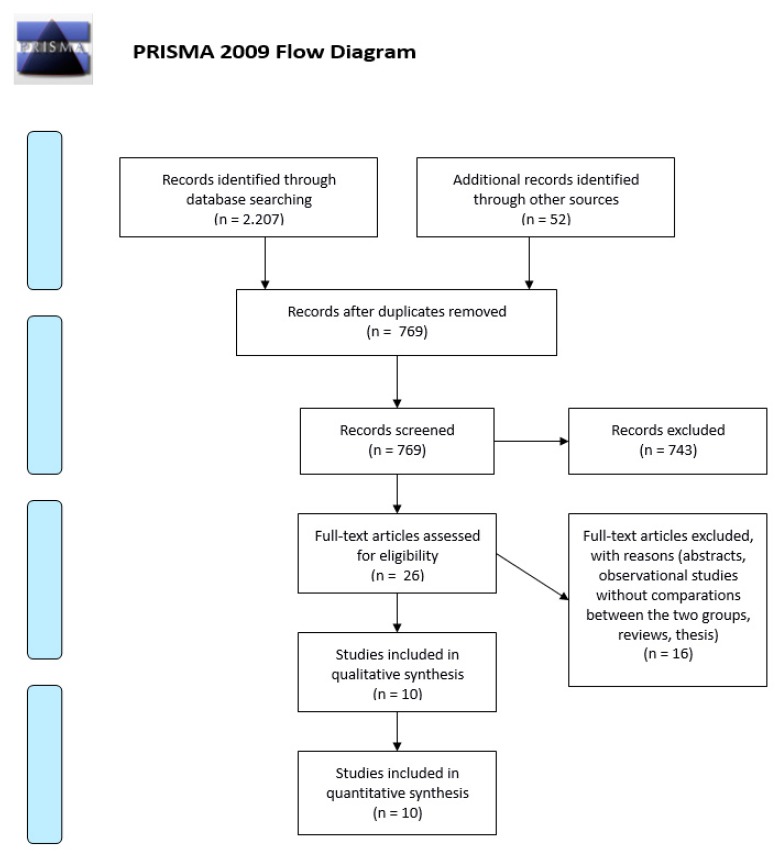
PRISMA flowchart of literature search.

The electronic search strategy identified 2259 citations (Figure 1). After the initial screening of the titles and abstracts and the removal of duplications, 26 papers remained [8,9,10,11,12,13,14,15,16,17,18,19,20,21,22,23,24,25,26,27,28,29,30,31,32,33]. After the evaluation of the full text, 16 studies were excluded [18,19,20,21,22,23,24,25,26,27,28,29,30,31,32,33] (Table A1, Appendix A). Only 10 studies met the inclusion criteria and were included in the analysis [8,9,10,11,12,13,14,15,16,17]. Four of them were performed in Europe and 6 were performed in the USA. The more extensive epidemiological studies were conducted in the USA (Table 1).

No definition of acute complicated diverticulitis was reported in any study. In seven studies, the codes for the inclusion of complicated diverticulitis were made explicit: Three articles reported ICD 9 and four studies reported ICD-10 (Table 2). In the selected database articles, only Diamant [11], Hupfeld [16], and Amato [17] described the proportion of hospitalizations for complicated AD– 41.6%, 14.8%, and 79.4%, respectively. The other studies reported absolute values, but it was not possible to evaluate the relative percentage (Table 3).

Some studies performed a distinction between abscesses and peritonitis, but it was not possible to distinguish the various types of abscess (Hinchey I vs. Hinchey II) and peritonitis (Hinchey III vs. Hinchey IV). These studies used the ICD 9-CM codes, and the distinction between stage I and stage II was only possible for code 569.5 (abscess of intestine) [9,11] (Hinchey I) and 614.3 (pelvic abscess) [11] (Hinchey II), while code 567.22 (peritoneal abscess) did not permit the distinction between stage I and stage II [11].

Regarding the peritonitis, codes 567.21 (generalized acute peritonitis) and 567.0 (peritonitis in infectious diseases classified elsewhere) were included, which allowed us to highlight the presence of peritonitis, but it did not enable us to distinguish Hinchey III from Hinchey IV [11,17]. The same was the problem with code K572C in ICD-10. The other used codes were 567.9 peritonitis (peritonitis unspecified) and 614.5 (acute or unspecified pelvic peritonitis, female), and they had the same limit. In some studies, the presence of perforation was reported, but the type of complication associated with perforation (abscess, peritonitis) was not described. In these studies, the code used was 569.83 (perforation of the intestine) [9,11,17]. In the group with peritonitis, based on the percent of all hospitalizations, Amato reported a higher rate of colonic perforation (10.2%) in contrast to Ricciardi (1.5%) and Rose (3.4%) (Table 4) [9,12,17].

Differently, the rate of diverticular abscess varied between 1.2% and 10% of all hospitalizations and from 5% to 29% of the hospitalized complicated cases (Table 5). Only Ricciardi et al. reported a temporal trend of the perforation rate in 1991 (start of study: 504, 1.6%) and 2005 (end of study: 910, 1.5%) and regarding the abscess rate of the same years (1991: 1855, 5.9% and 2005: 5837, 9.6%) [9]. Similarly, only one study reported a subgroup difference in the abscess rate in purulent and fecal peritonitis—4.5% and 29%, respectively [8].

Only Rose [12] and Amato [17] reported the presence of sepsis identified with the codes 785.52, 995.90–995.92, intestinal obstruction, and fistulas with the codes 569.81, 596.1, and 619.1. On the contrary, in the ICD-10 classification, the only codes concerning the complicated AD were the code K57.2 for diverticulitis of large intestine with perforation and abscess [13,15] and the code K566 for colonic stenosis (Table 2). Hupfeld et al. included all ICD-10 diagnosis codes but did not report a subgroup analysis of these codes [16].

## 4. Discussion

Nowadays, the colonic diverticular disease has a high impact on the resources of the National Health Service. Due to the increasing prevalence of this condition, the incidence of diverticular disease is increasing over time in both Western and Asian countries [34,35].

Currently, there is little information on the epidemiology of complicated AD, with most data deriving from the American health system, which significantly differs from the European systems, which are health national or mutual insurance. In Italy, the health system used is Beveridge-type, and the only studies reported in the literature using this system were carried out in England and Denmark. However, these studies were very few because they were carried out in only some regions or hospitals. We performed an analysis of the international and national administrative databases concerning admissions for complicated AD to update the epidemiology data.

The first obstacle in this regard is the lack of uniform definition for complicated AD in literature and everyday clinical practice. The most common was the presence of “diverticulitis with phlegmon, abscess, fistula, stricture or peritonitis” [36]. Many classifications of the complicated AD—clinical, radiological, or mixed—have been proposed in the literature [37].

In 1978, the first classification, proposed by Hinchey, categorized the patients into pericolic abscess or phlegmon confined to the mesentery of the colon (stage I), pelvic, intraabdominal, or retroperitoneal abscess resulting from a local perforation of a pericolic abscess (stage II), generalized purulent peritonitis resulting from rupture of pericolic/pelvic abscess into the peritoneal cavity (stage III), and generalized fecal peritonitis resulting from the free perforation of a diverticulum (stage IV) [38,39]. Successively, Wasvary et al. modified the Hinchey classification and suggested a difference between limited pericolic inflammation or phlegmon (stage Ia) and a confined pericolic abscess (stage Ib) [40]. In the same year, the consensus statement of the European Association of Endoscopic Surgeons (EAES) reported the new clinical classification of AD: Symptomatic disease, recurrent symptomatic illness, and complicated disease. Complicated AD included hemorrhage, fistula, phlegmon, abscess, purulent and fecal peritonitis, perforation, small bowel obstruction due to post-inflammatory adhesions, and stricture [41].

We made a large selection of studies according to the cited exclusion criteria and inclusion criteria to provide an objective analysis. We included only the databases focusing on the complicated AD and analyzed the coding systems (ICD 9-CM and ICD-10). In most of the included studies, it was not possible to derive the percentage of hospitalization for complicated AD because of the heterogeneity of the coding system. It is not possible to distinguish Hinchey III from Hinchey IV, while Hinchey I with code 569.5 was differentiated from Hinchey II with code 614.3, but the code 567.22 instead did not allow distinction (peritoneal abscess) in ICD-9-CM.

The Nationwide inpatient sample (NIS) data from 1998–2005 revealed a significant increase, with 26% of the hospitalizations for AD in the USA. The rate of surgery, however, decreased from 17.4% to 14.4% on the background of stable rate of colostomy (56%) and limited use of percutaneous drainage (1.4% to 2.5%) [5]. In another NIS survey encompassing the period 1991–2005, Ricciardi et al. demonstrated that the rate of admissions for AD was only 0.6% of all entries with increase of the ratio of the discharged diverticulitis from 5.1/1000 hospitalized patients in 1991 to 7.6/1000 in 2005. The rate of perforating disease remained stable (1.6% and 1.5% of all AD), whereas an increase from 5.9% to 9.6% for abscess was noted [9]. Extending the investigated period to 2008 (also NIS survey), Diamant et al. reported an 8% rate of abscesses and a similar rate of 1.6% perforating AD [11]. Based on the data of the California Office of Statewide Health Planning and Development Patient discharge database (PDD), Rose et al. also demonstrated an 8% abscess rate (27% of the complicated AD) and a 3.4% rate for peritonitis (11.5% of all AD admissions) for the period 1995–2009 [12].

In contrast to the abovementioned US studies, Amato et al. reported a significantly lower abscess rate (1.2%) and a higher rate of perforating AD—10.2% of all AD admissions and 41.6% of the complicated AD [17].

The somewhat confusing and inconclusive data represent a significant limitation of the present study and stems from the constraints of the included studies. The principal limit was the absence of ICD codes that permit to distinguish some different type of complications (Hinchey I vs. Hinchey II or Hinchey III vs. Hinchey IV) and often the lack of coding data about some general conditions such as sepsis, shock, malnutrition, steroid therapy, diabetes, and pulmonary and/or heart failure. Last, it should be noted that no uniform definition for complicated AD was used in the included studies. Additionally, the exact rate of the complicated AD admissions remains unknown because of the different healthcare systems and, particularly, the increasing trend for outpatient treatment of the uncomplicated and even selected complicated cases, which influences the denominator [42].

## 5. Conclusions

In general, the reported rates of abscess varied between 1–10% from all admissions for AD and 5–29% of the complicated AD. The diffuse peritonitis accounted for 1.6–10% of all hospitalizations and 11–47% of the complicated cases.

However, the available data precluded the definitive conclusions because of the significant discrepancy between the included studies. The main reason was the presence of coding inaccuracies in all databases and the absence of ICD codes that permitted us to distinguish some different types of complications (Hinchey I vs. Hinchey II or Hinchey III vs. Hinchey IV). Last, it should be noted that no uniform definition for complicated AD was used in the included studies. Nevertheless, the presented results and issues could be a starting point for future studies. Two suggestions could be made and discussed at future meetings. The first is the subdivision of the codes for purulent and feculent peritonitis in ICD-10. The second is the use of a strict definition of complicated AD and broader application of the World Society of Emergency Surgery (WSES) classification of AD, mainly because it is Computed tomography (CT)-based and allows staging even in the absence of operation in contrast to Hinchey [43].

## Figures and Tables

**Table 1:** Characteristics of the included studies.

Study	Years of the Research	Nation	N. of Patients		
Morris [8] 2008	1995–2000	The counties of Norfolk and Suffolk, UK	202 (only perforated diverticulitis)		
Ricciardi [9] 2009	1991–2005	USA	685,390		
Masoomi [10] 2011	2002–2007	USA	1,073,397		
Diamant [11] 2015	1993–2008	United States	822,865		
Rose [12] 2015	1995–2009	USA	210,268		
Hong [13] 2015	2009–2013	Australia	2829		
Lamm [14] 2016	1995–2014	USA	265,724		
Hong [15] 2017	2008–2014	USA, England, Australia	USA	England	Australia
			5332	6647	3171
Hupfeld [16] 2018	2000–2012	Denmark	44,160		
Amato [17] 2019	2008–2015	Italy	41,622		

**Table 2:** The diagnosis coding system used in the included studies.

Study	General Codes for AD	Codes for Complicated AD		
Morris [8] 2008	NR	ICD-10:		
		K57.2 (perforation, abscess, or peritonitis of large intestinal diverticula), K57.4 (perforation, abscess, peritonitis of both small and large bowel diverticula) K57.8 (perforation, abscess, peritonitis of either small or large bowel diverticula)		
Ricciardi [9] 2009	ICD-9-CM: 562.11 and 562.13 (diverticulitis with and without mention of hemorrhage)	ICD-9-CM		
		569.83 (diverticular free perforation) 569.5 (abscess)		
Masoomi [10] 2011	ICD-9-CM: 562.11 and 562.13 (diverticulitis with and without mention of hemorrhage)	NR		
Diamant [11] 2015	ICD-9-CM:562.11 and 562.13 (diverticulitis with and without mention of hemorrhage)	ICD-9-CM:		
		567.22, 569.5 and 614.3: Pericolic, mesenteric, or walled-off pelvic abscess (Hinchey I or II) 567, 567.21, 567.9 and 614.5: Generalized purulent or fecal peritonitis (Hinchey III or IV)		
Rose [12] 2015	ICD-9-CM: 562.11 and 562.13 (diverticulitis with and without mention of hemorrhage)	NR		
Hong [13] 2015	NR	ICD-10-AM:		
		K57.22 (diverticulitis with perforation and abscess, without hemorrhage) Similarly, due to lack of a specific diverticular K56.6 (stricture, we defined symptomatic stricture as admissions with large bowel obstruction in conjunction with diverticular disease (K57) and requiring an operation.		
Lamm [14] 2016	ICD-9-CM: 562.11 and 562.13 (diverticulitis with and without mention of hemorrhage)	NR		
Hong [15] 2017	NR	USA	England ICD-10	Australia ICD-10-AM
			K57.2	K57.2
Hupfeld [16] 2018	ICD-10 uncomplicated AD:	ICD-10:		
	K573 (diverticulosis or diverticulitis in the colon without perforation/abscess) K573A (diverticulitis in the colon without perforation) K573B (diverticulitis in the colon not otherwise specified)	K572 (diverticulosis or diverticulitis in the colon with perforation/abscess) K572A (diverticulitis in the colon with abscess) K572B (diverticulitis in the colon with perforation) K572C (diverticulitis in the colon with peritonitis)		
Amato [17] 2019	ICD-9-CM: 562.11 and 562.13 (diverticulitis with and without mention of hemorrhage)	ICD-9-CM:		
		560.0, 560.1, 560.2, 560.89, 560.9 (intestinal obstruction) 567.0-567.3, 567.9 (peritonitis) 578.0-578.9 (diverticular bleeding) 569.5 (intestinal/peritoneal abscess) 569.81 (intestinal fistula) 596.1 (colovescical fistula) 569.83 (intestinal perforation) 785.52, 995.90, 995.92 (sepsis or septic shock)		

**Table 3:** Patients hospitalized for complicated acute diverticulitis (AD).

Study	Number or Percentage			Total Patients		
Morrison [8] 2008 (in 5 years)	202			NR		
Ricciardi [9] 2009 (in 14 years)	NR			685,390		
Masoomi [10] 2011 (in 5 years)	840,157			1,073,397		
Diamant [11] 2015 (in 15 years)	79.4%			822,865		
Rose [12] 2015 (in 14 years)	61,064			210,268		
Hong [13] 2015 (in 4 years)	724			2829		
Lamm [14] 2016 (in 19 years)	NR			265,724		
Hong [15] 2017 (in 6 years)	USA	England	Australia	USA	England	Australia
	1729	1677	771	5332	6647	3171
Hupfeld [16] 2018	485 patients (12.98%) (in 2000) vs. 692 patients (14.83%) (in 2012)			44,160		
Amato [17] 2019	41.62%			174,436		

**Table 4:** The rate of generalized purulent or fecal peritonitis in patients with complicated AD.

Study	N of Peritonitis	N of Hospitalizations for Complicated AD			% Hospitalizations for Complicated AD	N of Admissions for AD			% Hospitalizations for AD
Morris [8] 2008	96 * (III)	202			47.5%	NR			NR
	38 ^†^ (IV)	202			18.8%	NR			NR
Ricciardi [9] 2009	504 ^‡^	NR			NR	685,390			1.6%
	910 ^§^	NR			NR				1.5%
Masoomi [10] 2011	NR	840,157			NR	1,073,397			NR
Diamant [11] 2015	NR	NR			NR	822,865			1.6% ^‖^
Rose [12] 2015	7044	61,064			11.5%	210.268			3.4%
Hong [13] 2015	NR	724			NR	2,829			NR
Lamm [14] 2016	NR	NR			NR	NR			NR
Hong [15] 2017	NR	USA	England	Australia	NR	USA	England	Australia	NR
		1729	1677	771		5332	6647	3171	
Hupfeld [16] 2018	NR	NR			NR	44,160			NR
Amato [17] 2019	17.811	41,622			42.79%	174,436			10.21%

* Perforation with diffuse purulent peritonitis, ^†^ Perforation with diffuse fecal peritonitis, ^‡^ 1991, ^§^ 2005, ^‖^ Hinchey III–IV.

**Table 5:** Abscess in patients with complicated AD.

Study	N of Abscess	N of Hospitalizations for Complicated AD			% Hospitalizations for Complicated AD	N of Admissions for AD			% Hospitalizations for AD
Morris [8] 2008	9 *	202			4.5%	NR			NR
	59 ^†^	202			29.2%	NR			NR
Ricciardi [9] 2009	1855 ^‡^	NR			NR	685,390			5.9%
	5837 ^§^	NR			NR				9.6%
Masoom [10] 2011	NR	840,157			NR	1,073,397			NR
Diamant [11] 2015	NR	NR			NR	822,865			8.1% ^‖^
Rose [12] 2015	16,613	61,064			27.2%	210,268			7.9%
Hong [13] 2015	NR	724			NR	2829			NR
Lamm [14] 2016	NR	NR			NR	NR			NR
Hong [15] 2017	NR	USA	England	Australia	NR	USA	England	Australia	NR
		1729	1677	771		5332	6647	3171	
Hupfeld [16] 2018	NR	NR			NR	44,160			NR
Amato [17] 2019	2143	41,622			5.15%	174,436			1.23%

* Perforation with pericolic abscess, ^†^ Perforation with intra-abdominal/pelvic abscess, ^‡^ 1991, ^§^ 2005, ^‖^ Hinchey I–II.

## Appendix A

**Table A1:** The list of the excluded studies.

Author and Year of Publication	Reason for the Exclusion
Cammarota 2018	There is overlapping with Amato 2019
Lee 2018	The authors compared the BMI, computed tomographic estimations of abdominal fat content, age, and sex
Dean 2018	The authors identified only patients ≤50 with a diagnosis of diverticulitis in the NSQIP database
Cirocchi 2018	The authors performed an anaòysis of surgical strategies in elderly patients
Mennini 2017	The authors reported an economic analysis of diverticular disease
Tan 2016	The authors performed an analysis of the predictors of acute diverticulitis severity
Jamal Talabani 2016	The authors performed an evaluation for risk factors for admission for acute colonic diverticulitis
Bharucha 2015	The authors performed a population-based analysis of acute diverticulitis
Schneider 2015	The authors performed an analysis of emergency department presentation, admission, and surgical intervention for colonic diverticulitis in the United States, but they do not report any data about the complicated diverticulitis
Jamal Talabani 2014	The authors performed a hospital analysis of patients with acute diverticulitis
Kim 2012	The authors reported an analysis of the clinical factors for predicting severe diverticulitis in Korea
Sorser 2009	The authors performed an analysis of the association between obesity and complicated diverticular disease
Andeweg 2008	The authors evaluated the incidence and risk factors of recurrence after surgery for acute diverticulitis
Papagrigoriadis 2004	The authors performed an analysis about clinical and cost analysis of inpatient and outpatient investigations, treatment and hospitalization
Kang 2003	The authors do not report any data about the complicated diverticular disease
McConnell	The authors performed a hospital analysis of patients with acute diverticulitis
